# Supplementary material for: Pathway information extracted from 25 years of pathway figures
Source: Genome Biol. 2020 Nov 9;21:273. doi: 10.1186/s13059-020-02181-2 (PMC7649569; doi:10.1186/s13059-020-02181-2)
Supplement: Supplementary file 1 — Additional file 1 Review history. [file 13059_2020_2181_MOESM1_ESM.docx]

**First round**

**Reviewer 1**

Hanspers, et al, describe an approach to systematically analyze and annotate published pathways diagrams into a queryable web based tool. This is a heroic undertaking that results in a useful tool that is likely to be of broad interest to the scientific community. The approach is sound, the methods are well-described, and a few use cases are represented. Overall, I think this manuscript makes a worthwhile contribution and would benefit from just a few minor revisions.

Major comments:

1. The search to identify pathways for inclusion in the database included cancer. The authors should comment on this in the discussion, as it is circular with the disease enrichment analysis presented in Fig 2.

2. The history of Hippo is kind of interesting, but I am not really sure of what we learn from this sort of history analysis. Is there a take-home message that could inform how we use pathway diagrams to understand new discoveries, or some insights into how we might speed up the process of dissecting/annotating new pathways?

Minor comments:

1. Include specific example figures of the various pathway categories to concretely describe the types of pathways diagrams that passed the filter (p.2)

2. Several comparisons to PubTator are made. A description of exactly what PubTator is and how the algorithm differs from the one presented here would increase clarity and make the paper accessible to a wider audience (p.3)

3. Mapping to gene names is a reasonable approach and well justified. However, there are many considerations for how this may be implemented. For example, how are molecules such as phosphor-AKT handled? Some additional details in the main text about this would be helpful (p.3)

4. It was not clear exactly what is meant by "unexpanded" genes and why this is important to consider - this is another case where a specific example could help to unpack the meaning (p.4)

5. Several details of the methods should be incorporated into main text to increase clarity:

a. Keyword search terms used to identify pathway figures

b. Specific definition of pathway (p.9-10)

c. Additional details about the OCR pipeline (p.10)

6. The Shiny app is quite nice, but I found it sometimes a bit slow to load. The authors should consider whether there may be ways to speed up the processing time.

**Reviewer 2**

This manuscript describes the construction of a database of gene names found in published pathway diagrams. Nearly 65,000 diagrams drawn to represent the interactions of proteins were collated from papers spanning the time period 1995 to 2019, and automated methods were used to identify gene names within each of these diagrams. The groups of gene names represented by each diagram have been made available through a web tool. The authors demonstrate a number of uses for this database by examining enrichments and trends found within these associations, such as the evolution of the Hippo pathway concept over time and the identification of a consensus core and peripheral genes.

Overall, I think this somewhat unusual paper clearly presents what is quite a useful resource that appears to be easily usable for a number of purposes. It is a significantly larger database than others that have preceded it, and it should be of broad interest across many molecular fields. While it is easy to imagine ways in which the authors could go further than they have in this manuscript, I don't see the need to request further additions to what is already a useful and functional resource. I just have a small number of minor comments.

1. As the authors point out, pathway diagrams contain a large amount of information, and the gene name associations gleaned here are really the tip of the iceberg. Much more untapped information lies in the various arrows, organelles, and spatial groupings that are included in many figures. While it is easy to understand that extracting this graphical information will be quite challenging, I think it is worth spending some more time discussing the prospects how such information might be collected and codified.

2. One puzzling aspect of the database is found in figure 2, where the disease terms associated with genes found in the figure diagrams is compared to the terms associated with the papers. As the authors point out, "The gene information provided by the pathway figures allows for enrichment against higher resolution disease ontologies". However the top 10 diseases found are not at all what one might expect - relatively rare diseases such as juvenile rheumatoid arthritis and primary hyperaldosteronism make prominent appearances while diabetes and cardiovascular disease do not. I don't quite follow the explanation for why the higher resolution leads to these particular identifications. I think a substantially more detailed explanation of why this occurs would be interesting and informative.

**Response**

**Reviewer 1**

Hanspers, et al, describe an approach to systematically analyze and annotate published pathways diagrams into a queryable web based tool. This is a heroic undertaking that results in a useful tool that is likely to be of broad interest to the scientific community. The approach is sound, the methods are well- described, and a few use cases are represented. Overall, I think this manuscript makes a worthwhile contribution and would benefit from just a few minor revisions.

Major comments: **1. The search to identify pathways for inclusion in the database included cancer. The authors should comment on this in the discussion, as it is circular with the disease enrichment analysis presented in Fig 2.**

Thank you for pointing this out. Our selection of query terms was driven by an independently-developed ontology of pathway types. But ontologies are not perfectly balanced nor free of bias in their construction, so your point still stands. We have added the following to the text:

*The prevalence of cancer in this set of papers is greater than that for PMC in general,* We have added a sentence to the main text where PubTator is first mentioned to make sure this distinction is clear.*which was estimated at 12%.[PMC5345838] This bias could reflect the popularity and particular effectiveness of pathway diagrams in depicting cancer-related signaling and metabolic processes. It could also result from a bias in the pathway ontology used to construct the original PMC query, which included "cancer pathways."*

2. The history of Hippo is kind of interesting, but I am not really sure of what we learn from this sort of history analysis. Is there a take-home message that could inform how we use pathway diagrams to understand new discoveries, or some insights into how we might speed up the process of dissecting/annotating new pathways?

Indeed, the specific details of the Hippo Pathway example are perhaps only interesting to researchers who care about the Hippo Pathway. But, we see the take-home messages as being generalizable: access to pathway diagrams and their gene contents provides a new way to track the discovery and depiction of key molecules and interactions for a given pathway over time; many of these pathway figures predate *any* pathway database. The example also demonstrates the utility of having access to this information *in a computational format* for the first time, enabling clustering, visualization and other applications. Additionally, the example illustrates a novel way to conceive of pathways as a hierarchically organized collections of core and peripheral components rather than as singular, canonical diagrams.

We have added text to the manuscript to clarify these messages and point out that they should generalize to a wide variety of well-studied pathways beyond just the Hippo Pathway.

Minor comments: 1. Include specific example figures of the various pathway categories to concretely describe the types of pathways diagrams that passed the filter (p.2)

It is difficult to concretely describe what is and is not a pathway. We trained a machine to learn how to make this distinction, but this does not make it any easier to describe in words. So, we agree with the reviewer that the images themselves present the best description. However, they do not fall into 3 or 4 simple categories. They are all over the place! I could attempt to pick 3 or 4 examples, but this could just

as easily lead to misconceptions of types that were not included. In the end, the sum total of 15,406 images went into our machine learning-based definition of pathway and non-pathway. These are specified by the “type.man” column in the supplemental file we are providing: https://nih.figshare.com/articles/dataset/65k_pathway_figures_published_between_1995_and_2019/1242 0887 This will allow anyone to more precisely determine what we considered a pathway in this work. We have added this note to the manuscript.

2. Several comparisons to PubTator are made. A description of exactly what PubTator is and how the algorithm differs from the one presented here would increase clarity and make the paper accessible to a wider audience (p.3)

The primary distinction is simply where we are looking. PubTator focuses on genes mentioned in the text, including the abstract and body of papers. We are performing OCR on images in order to identify genes mentioned in figures. Not the figure caption text, but the actual figure images. We have added a sentence to the main text where PubTator is first mentioned to make sure this distinction is clear.

The process of matching words found in text (original text or OCR’d text) is relatively the same, though we have optimized our pipeline for OCR-based text and pathway figures. This has also been added to the appropriate section and is described in more detail in the next response.

3. Mapping to gene names is a reasonable approach and well justified. However, there are many considerations for how this may be implemented. For example, how are molecules such as phosphor- AKT handled? Some additional details in the main text about this would be helpful (p.3)

Our process was called “Curated Optimization” and is described in an earlier methods paper on a sample of 4000 figures [https://doi.org/10.1101/379446]. We manually checked false positive and false negative matches between the OCR’d text and our lexicon of gene symbols. We then developed custom transforms to catch these cases. For example, we found numerous cases of gene symbols prefixed and suffixed with things like, p-, -FLAG, -WT, etc. We then developed a match/substitution script to replace these. Other transforms focused on common OCR mistakes, for example with Greek characters or with confusing capital I, lowercase l and the number one. These transforms are critical to the pipeline. We have added this description and pointer to our code repository to the manuscript.

4. It was not clear exactly what is meant by "unexpanded" genes and why this is important to consider - this is another case where a specific example could help to unpack the meaning (p.4)

Our goal was to avoid counting *each member* of a protein complex or family when setting certain count- based thresholds, so that when we wanted figures with say 7 or more genes, we didn’t end up with a figure of a single complex (or family) that happened to have a lot of functionally-redundant members. This was a concern for the project early on, but in the end, turned out to not be a major source of bloated gene counts or other problems. So, we have changed the manuscript to remove the “unexpanded” terminology and to simply use the term, “distinct.”

5. Several details of the methods should be incorporated into main text to increase clarity:

In general, we feel that in the process of reformatting the manuscript for the Database article type the section formerly called Methods (and rendered in smaller print by some journals) is now more visible as the Construction section. So perhaps the contained details do not require repeating in the “main text.” Additional points per item are made below.

a. Keyword search terms used to identify pathway figures

These terms are currently provided in the third sentence of Construction: Collection of Figures. There we have space to explain where they are from and how we came to settle upon them, as well as provide an associated “Limitations” discussion. Listing the terms outside of this context may raise more questions than it answers.

b. Specific definition of pathway (p.9-10)

As described above, this is a subtly challenging task. The changes made above cover our response here as well.

c. Additional details about the OCR pipeline (p.10)

We added more details about transforms in a section that cites prior work and points to our code repository.

6. The Shiny app is quite nice, but I found it sometimes a bit slow to load. The authors should consider whether there may be ways to speed up the processing time.

We have increased the memory allocated to the instance and tuned various worker load parameters, but there isn’t much room for additional optimization using this platform. We might explore alternative technologies if user demand grows.

We are also pursuing a variety of hosts for the content, tailoring it to specific use cases, for example NDEx (for enrichment and network modeling use cases) and other literature search examples already mentioned. The NDEx example has been added to the manuscript.

**Reviewer 2**

This manuscript describes the construction of a database of gene names found in published pathway diagrams. Nearly 65,000 diagrams drawn to represent the interactions of proteins were collated from papers spanning the time period 1995 to 2019, and automated methods were used to identify gene names within each of these diagrams. The groups of gene names represented by each diagram have been made available through a web tool. The authors demonstrate a number of uses for this database by examining enrichments and trends found within these associations, such as the evolution of the Hippo pathway concept over time and the identification of a consensus core and peripheral genes. Overall, I think this somewhat unusual paper clearly presents what is quite a useful resource that appears to be easily usable for a number of purposes. It is a significantly larger database than others that have preceded it, and it should be of broad interest across many molecular fields. While it is easy to imagine ways in which the authors could go further than they have in this manuscript, I don't see the need to request further additions to what is already a useful and functional resource. I just have a small number of minor comments.

1. As the authors point out, pathway diagrams contain a large amount of information, and the gene name associations gleaned here are really the tip of the iceberg. Much more untapped information lies in the various arrows, organelles, and spatial groupings that are included in many figures. While it is easy to understand that extracting this graphical information will be quite challenging, I think it is worth spending some more time discussing the prospects how such information might be collected and codified.

Indeed, we are excited to continue mining this content (and the continuing stream of new pathway figure content) for metabolites, state information, and other biological concepts. As the reviewer notes, the detection of interactions (arrows), organelles and spatial groupings is a particular challenge that does not have a clear solution today. While we do have a handle on spatial information for the OCR’d text, the representation of interactions and organelles is far from standardized in practice and would likely involve custom machine learning approaches well beyond what we have employed here. We can at least offer our refined collection of figures as novel reference resource for those attempting this work. We have added this explicit offer to the manuscript.

To put a finer point on the challenge, the process of properly modeling interactions, organelles, protein states and complex membership involves much more than simply retracing what is typically provided in a figure diagram. So, even with high fidelity capture of location information and inferred interaction semantics, further processing (i.e., by a domain expert) would be required to generate sufficiently accurate models for computational analysis. We have added these points to the manuscript discussion as well. Thank you for raising them.

**2. One puzzling aspect of the database is found in figure 2, where the disease terms associated with genes found in the figure diagrams is compared to the terms associated with the papers.** As the authors point out, "The gene information provided by the pathway figures allows for enrichment against higher resolution disease ontologies". However the top 10 diseases found are not at all what one might expect - relatively rare diseases such as juvenile rheumatoid arthritis and primary hyperaldosteronism make prominent appearances while diabetes and cardiovascular disease do not. I don't quite follow the explanation for why the higher resolution leads to these particular identifications. I think a substantially more detailed explanation of why this occurs would be interesting and informative.

We appreciate this observation and share your concern for exactly how to present these disease annotations. In the end, we are comparing very different things: (1) disease terms manually associated with papers by authors or biocurators and (2) disease terms represented by curated gene sets that algorithmically enrich for one or more pathway figures via our extracted genes. Furthermore, the disease terms are from two different ontologies and the relevant subsets of paper handled by each case are different. We have decided to remove this comparison from the text and as Figure 2.

The updated manuscript still presents the paper annotations, but now presents the characterization of extracted gene sets from figures as a separate matter. Focusing on the latter case, we manually verified a subset of the disease enrichment results in order to weed out the false positive hits one routinely gets from overrepresentation analysis. We present the verified annotations, which indeed align better with expectations. Thanks again for your critique.
